# Comparison of oral glucose tolerance test and HbA1c in detection of disorders of glucose metabolism in patients with acute stroke

**DOI:** 10.1186/s12933-020-01182-6

**Published:** 2020-12-05

**Authors:** Karl Matz, Jaakko Tuomilehto, Yvonne Teuschl, Alexandra Dachenhausen, Michael Brainin

**Affiliations:** 1 Department for Clinical Neurosciences and Preventive Medicine, Danube University Krems, Krems, Austria; 2 Department of Neurology, Landesklinikum Baden-Mödling, Mödling, Austria; 3 Department of Public Health, University of Helsinki, Helsinki, Finland; 4 Public Health Promotion Unit, Finnish Institute for Health and Welfare, Helsinki, Finland; 5 National School of Public Health, Madrid, Spain; 6 Diabetes Research Group, King Abdulaziz University, Jeddah, Saudi Arabia

**Keywords:** Risk assessment, Fasting glucose, HbA1c, Oral glucose tolerance test, Acute stroke, Type 2 diabetes, Glucose abnormalities

## Abstract

**Background:**

Diabetes is an increasingly important risk factor for ischemic stroke and worsens stroke prognosis. Yet a large proportion of stroke patients who are eventually diabetic are undiagnosed. Therefore, it is important to have sensitive assessment of unrecognized hyperglycaemia in stroke patients.

**Design:**

Secondary outcome analysis of a randomized controlled trial focussing on parameters of glucose metabolism and detection of diabetes and prediabetes in patients with acute ischemic stroke (AIS).

**Methods:**

A total of 130 consecutively admitted patients with AIS without previously known type 2 diabetes mellitus (T2DM) were screened for diabetes or prediabetes as part of secondary outcome analysis of a randomized controlled trial that tested lifestyle intervention to prevent post-stroke cognitive decline. Patients had the oral glucose tolerance test (OGTT) and glycated hemoglobin (HbA1c) measurements in the second week after stroke onset and after 1 year. The detection rates of diabetes and prediabetes based on the OGTT or HbA1c values were compared.

**Results:**

By any of the applied tests at the second week after stroke onset 62 of 130 patients (48%) had prediabetes or T2DM. Seventy-five patients had results from both tests available, the OGTT and HbA1c; according to the OGTT 40 (53.3%) patients had normal glucose metabolism, 33 (44%) had prediabetes, two (2.7%) T2DM. In 50 (66.7%) patients the HbA1c results were normal, 24 (32%) in the prediabetic and one (1.3%) in the diabetic range. The detection rate for disorders of glucose metabolism was 10% higher (absolute difference; relative difference 29%) with the OGTT compared with HbA1c. After 1 year the detection rate for prediabetes or T2DM was 7% higher with the OGTT (26% relative difference).

The study intervention led to a more favourable evolution of glycemic status after 1 year.

**Conclusion:**

The OGTT is a more sensitive screening tool than HbA1c for the detection of previously unrecognized glycemic disorders in patients with acute stroke with an at least a 25% relative difference in detection rate. Therefore, an OGTT should be performed in all patients with stroke with no history of diabetes.

* Trial registration* http://clinicaltrials.gov. Unique identifier: NCT01109836.

## Background

Glucose abnormalities, either diabetes type 2 (T2DM) or impaired glucose tolerance (IGT) are common in patients with stroke and impair their prognosis [1–5]. T2DM and IGT should therefore be included in the risk assessment of stroke patients. We have earlier shown that a high proportion of stroke patients with T2DM and all cases of IGT are undetected prior to the stroke event and would still remain undiscovered if fasting plasma glucose (FPG) alone is determined in the clinical evaluation of stroke patients [6].

It is a longstanding paradox that the OGTT is used for diabetes diagnosis whereas HbA1c serves as monitoring parameter for antidiabetic treatment. As the recommended 2-h oral glucose tolerance test (OGTT) may be a time-consuming procedure, an International Expert Committee and the American Diabetes Association (ADA) have recommend the use of HbA1c as a diagnostic test [7] with ≥ 6.5% (48 mmol/mol) as the diagnostic cut-point for T2DM. Also, the World Health Organisation (WHO) Consultation Group has agreed on the use of this HbA1c cut point but stressed that the OGTT should remain as the primary method to diagnose T2DM [8]. With the HbA1c in the guidelines, the OGTT is used less in the routine clinical practice although it is the only way to detect IGT as a postprandial dysregulation of blood glucose.

The aim of the present study was to find out how well HbA1c results will compare with the determination of FPG and 2-h plasma glucose (2hPG) using an OGTT in screening for T2DM and prediabetes in patients with acute non-disabling ischemic stroke during the early post-acute period and after the 1-year follow-up.

## Methods

In this study we performed a retrospective analysis of the data regarding glucose metabolism that were collected as part of the protocol in the Austrian Polyintervention Study to Prevent Cognitive Decline after Ischemic Stroke (ASPIS) trial. The study details of ASPIS have been described earlier [9]. In short, the ASPIS trial tested whether a 24-month intensified multidomain intervention, which targeted modifiable risk factors, can prevent post-stroke cognitive decline compared with a control group receiving standard care. A total of 199 patients with acute ischemic stroke during the last three months, aged 40 to 80 years, with a modified Rankin scale (mRS) of 0–2 on admission, and sufficient communication ability were enrolled from five stroke clinics in Austria between 2010 and 2012.

Patients were tested for diabetes or prediabetes with HbA1c or an OGTT at baseline in the first or second week after the stroke and at 1-year follow-up. Blood samples for FPG and 2hPG were taken in sodium fluoride tubes and analysed immediately with the hexokinase method in a Hitachi Laboratory systems machine. The World Health Organization (WHO) 1999 criteria for diabetes [10] (either FPG ≥ 7.0 mmol/l or 2hPG ≥ 11.1 mmol/l), impaired fasting glucose (IFG) (FPG 6.1–6.9 mmol/l and 2hPG < 7.8 mmol/l) and IGT (2hPG 7.8–11.0 mmol/l) were used. Those whose FPG and 2hPG were below these levels were considered to have normal plasma glucose. HbA1c was determined from whole blood samples by turbidimetric inhibition immunoassay. HbA1c 48 mmol/mol (6.5%) was considered as the cut-point for diabetes, and a range between 5.7 and 6.4% as prediabetic. Patients with known T2DM did not undergo OGTT and were excluded from this analysis.

We further assessed if glucose metabolism improved, stayed stable or worsened between baseline and follow-up. Worsening was defined as progression from normal glucose metabolism to prediabetes or T2DM or from prediabetes toT2DM, improvement as change from T2DM to prediabetes or to normal metabolism or from prediabetes to normal.

### Statistical analyses

Observed agreement between the diagnostic methods were calculated as percentage ratio between overlapping diagnosis and overall numbers of cases with disease. Concordance of methods was calculated as Cohens’ kappa coefficient.

Group comparisons between the improving or stable and worsening group were calculated with Chi-square test for categorical variables and with Kruskal–Wallis’ test for numeric variables. The level of significance was set two sided at α = 0.05. Statistical analyses were carried out with IBM SPSS Statistics 26.0.

## Results

**Fig. 1:**
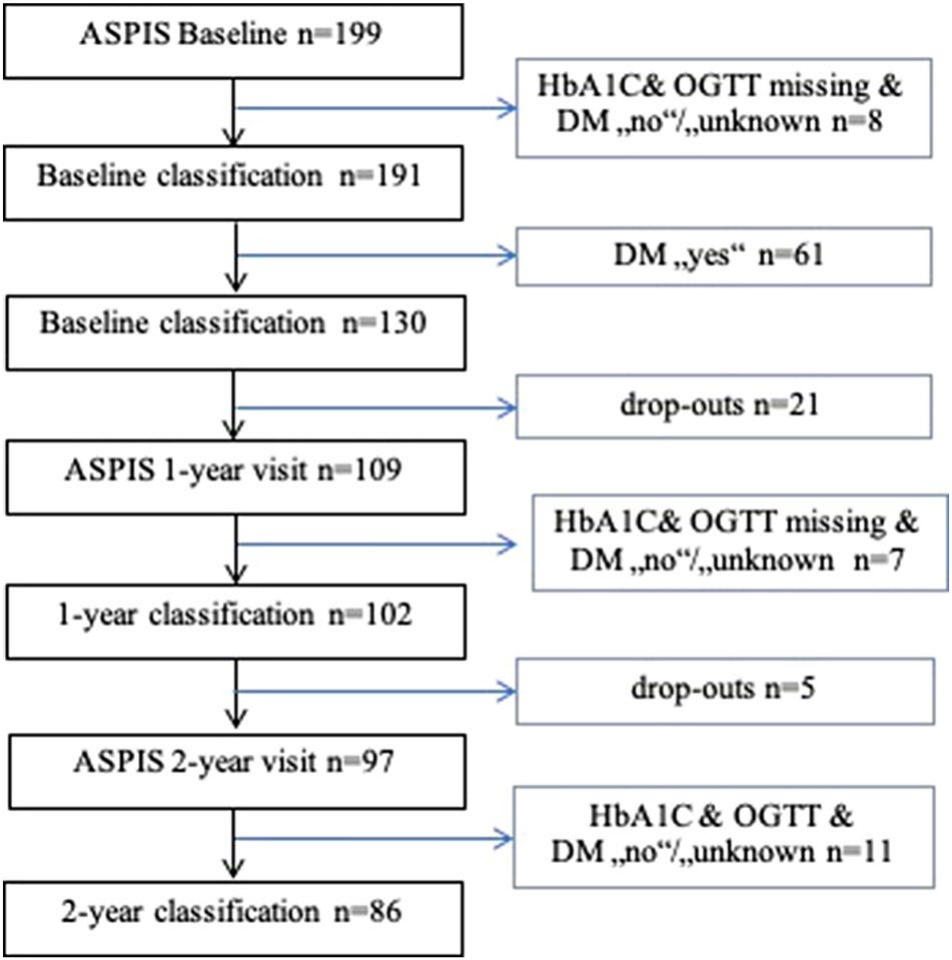
CONSORT flow diagram of classification for disorders of glucose metabolism during the ASPIS randomized controlled trial

Of the 199 patients included in the ASPIS trial, 61 (30.7%) had already T2DM according to patients’ history, eight patients without previous diagnosis had no results for HbA1c or the OGTT available to allow classification at baseline, leaving 130 patients for further analysis (see Fig. 1). The results of the baseline classification were as follows: five patients (3.8%) without a prior diabetes diagnosis were found to have diabetes either by HbA1c or the OGTT results, prediabetes was found in 57 (43.8%) patients, and 68 patients had normal test results (52.3%).

Both the OGTT and HbA1c results were available in 75 patients. According to the OGTT 40 (53.3%) patients had normal glucose metabolism, 33 (44%) had prediabetes, two (2.7%) T2DM. According to the HbA1c results 50 (66.7%) were normal, 24 (32%) prediabetic and one T2DM (1.3%), see Table 1. The observed agreement in diagnosis of T2DM was 50%, of prediabetes only 42.4%. Cohens’ Kappa coefficient for concordance of HbA1c and the OGTT was 0.205. The detection rate for disorders of glucose metabolism was 16.7% higher (absolute difference; relative difference 28.6%) with the OGTT than with HbA1c.

**Table 1:** Cross table of resulting classifications by OGTT and HbA1c in patients who had both tests at baseline (n = 75) and 12 months follow up (n = 67)

Baseline	HbA1c		
	Normal	Prediabetes	Diabetes
OGTT			
Normal	30	10	0
Prediabetes	19	14	0
Diabetes	1	0	1

12 months	HbA1c		
	Normal	Prediabetes	Diabetes
OGTT			
Normal	37	8	0
Prediabetes	13	6	0
Diabetes	1	2	0

After 1 year, 28 patients were either study dropouts or had no further tests for glucose metabolism available, leaving 102 subjects for the follow-up analysis: the results of either tests revealed 56 patients as normal (54.9%), 36 as prediabetes (35.3%) and 10 (9.8%) with T2DM (three according to OGTT, one according to HbA1c and six according to updated clinical information).

67 (66%) patients had both tests, OGTT and HBA1c, available for direct comparison: according to OGTT 45 (67.2%) patients had normal glucose metabolism, 19 (28.4%) had prediabetes, and three (4.5%) T2DM. According to HbA1c results 51 (76.1%) were normal and 16 (23.9%) prediabetic. The observed agreement in classification of normal glucose metabolism was 62.7%, of prediabetes only 20.7%, the detection rate for prediabetes or T2DM was 9.9% (absolute difference; 30% relative difference) higher with OGTT, (see Table 1). Cohens’ Kappa coefficient dropped to 0.149.

**Fig. 2:**
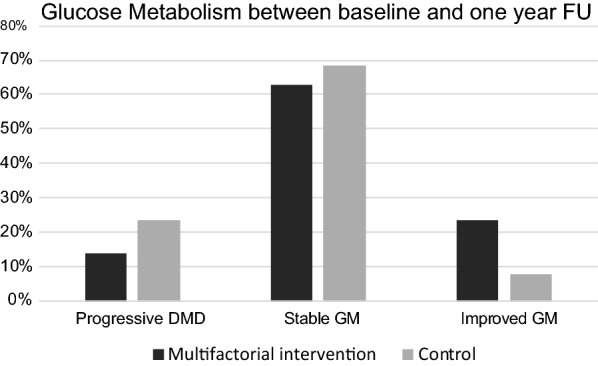
Percentage of progressive diabetic metabolic disorder (DMD), stable or improved glucose metabolism (GM) between baseline and 1 year follow up in the multifactorial intervention group of the ASPIS trial and in controls. Both, OGTT and HbA1c were used for diagnosis, patients were classified to PD or T2DM if either the OGTT or HbA1c criteria were fulfilled. Progressive DMD was defined as progression from normal GM to PD or T2DM or from PD to T2DM, improved GM as change of GM in the opposite direction

Next we looked how glycemic status changed over time in patients who had tests both at baseline and 1-year follow-up: Out of 102 patients 67 (65.7%) remained stable, 16 (15.7%) improved and 19/102 (18.6%) progressed to prediabetes or T2DM. Baseline variables of these three groups are shown in Additional file 1: Table S1. In addition, we compared these groups separately according to their allocation to intervention or control group in the randomized controlled trial: In the intervention group seven (13.7%) of 51 patients showed progression to prediabetes or T2DM, in the control group 12 (23.5%) of 51 patients (p = 0.066). 12 patients (23.5%) in the intervention group improved their glucose metabolism compared to only four (7.8%) in the control group (see Fig. 2).

## Discussion

### Summary

The main finding of this study was that in patients which subacute minor stroke the OGTT detects up to 25 to 29% more patients with prediabetes or T2DM than HbA1c regardless if performed early after the event or at 1-year follow-up. The odds that prediabetes was detected by the OGTT was 2.6 higher compared to HbA1c measurement alone. As in several previous studies [9, 11, 12] disorders of glucose metabolism were highly prevalent (more than 60%) in patients with AIS. Overall, the concordance between the OGTT and HbA1c results was low, which has also been found by several other studies [13, 14].

During the 1-year observation the classification of glucose disorders in stroke patients in the trial was not invariable, although about two thirds of the patients did not change their original category. A multifactorial lifestyle-oriented intervention resulted in a non-significant statistical trend for a higher percentage of patients who improved in their glucose metabolism in the intervention group than the control group receiving a standard care. With improvements in lifestyle [15] or treatment with metformin [16], acarbose [17], pioglitazone [18] or liraglutide [19] the risk of the conversion from prediabetes to frank T2DM can be reduced. Detection of diabetes and prediabetes is also of importance for secondary prevention of ischemic stroke, for example pioglitazone [20] reduces the risk of stroke recurrence in stroke patients with prediabetes or T2DM.

### Diagnostic advantage of OGTT

American Diabetes Association has recommended HbA1c as the tool for diagnosing T2DM and defined a cut-off level 48 mmol/mol to indicate diabetes [7]. Since long-term hyperglycaemia is necessary to raise HbA1c to this level (when other causes of HbA1c elevation are excluded), HbA1c above this cut point obviously has a high specificity. Due to this high specificity, also WHO has agreed that HbA1c 48 mmol/mol (6.5%) or above can be used to diagnose diabetes, but WHO still gives preference to the determination of glucose and use of the OGTT due to the superior sensitivity [8]. The sensitivity of HbA1c alone is low leaving many people with T2DM and all people with IGT undetected. However, screening for prediabetes is especially important in patients with already treated other vascular risk factors [21]. Therefore, the WHO report emphasizes that HbA1c value below 48 mmol/mol does not exclude diabetes diagnosed using glucose tests, and that there is insufficient evidence to make any formal recommendation on the interpretation of HbA1c levels below 48 mmol/mol. Our results in acute stroke patients provides a very strong support to this statement.

### Prognostic advantage of OGTT

There is also evidence that the parameters of the OGTT correlate better with outcomes of vascular disease than HbA1c. A German study among 1015 patients admitted for coronary angiography found T2DM in 149 patients by an OGTT, but only 42 by HbA1c [22] and the severity of coronary heart disease (CHD) correlated with 2hPG in the OGTT. This is in keeping with the data from previous studies that have shown that 2hPG is the best predictor of the three glycaemic parameters for incident cardiovascular events [23], total mortality [24] or intima-media thickness of carotid arteries [25]. Postprandial glycemic peaks seem also lead to maladaptive vascular remodelling [26]. Several studies in the general population have confirmed that 2hPG is a predictor of cardiovascular outcome independent of FPG and HbA1c [27–29]. In the EUROSPIRE-IV survey [30] 2 h-PG in contrast to HbA1c was an independent predictor of subsequent new cardiovascular events in a large cohort of patients with CHD. Both HbA1c and 2 h-PG were independent predictors of incident new T2DM during follow-up. Furthermore, in a Chinese study newly diagnosed T2DM was independently associated with 1-year mortality, functional outcome and stroke recurrence [31]. In our previous observational study we also showed that undiagnosed T2DM may worsen the prognosis in stroke patients [6].

### Strengths

The strength of our study is that we ascertained consecutively patients admitted that were assessed and reassessed within the settings of a randomized controlled trial. Secondly, we performed a follow-up up investigation after 12 months and therefore our results do not rely only on measurements during the acute stroke period.

### Limitations

Weak points of our study are the low number of patients investigated as this was a regionally conducted multi-centre trial and the considerable number of dropouts. Despites this weakness in quantity our results are robust over time from baseline to follow up and confirmatory with other studies. Furthermore, it can be stated that part of the hyperglycaemic states that were classified as diabetes and prediabetes at baseline can be interpreted as acute hyperglycaemic stress responses [32] especially in severe strokes and that the OGTT is more sensitive to these acute fluctuations of blood glucose levels. However, we still observed the difference between OGTT and HbA1c after 1 year when stress induced hyperglycaemia was no longer existing, and the influence of stroke severity was probably low as mostly minor strokes were included into the trial. Recently a study [33] found that several parameters referring to the metabolic syndrome fairly predicted persistent IGT in patients with acute minor stroke.

In addition, we found a trend for a lower rate of progression of disorders of glucose metabolism among those who underwent multimodal interventions for risk factor control compared with the control group. An improved detection of prediabetes and early T2DM using an OGTT could help to focus and tailor preventive interventions to improve or maintain cardiovascular and metabolic health.

## Conclusion

In the clinical evaluation of patients with acute stroke the assessment of the glycemic status is important in order to assign adequate preventive management to correct metabolic abnormalities that may influence the prognosis. The OGTT is a more sensitive method than HbA1c to detect and monitor disorders of glucose metabolism in patients with an ischemic stroke. The OGTT should be carried out in all stroke patients who have not been found to be diabetic before the stroke event.

## Supplementary Information

**Additional file 1: Table S1.** Baseline characteristics of patients in different groups of evolution of glucose metabolism (GM) between baseline and 12 months follow up. Both, OGTT and HbA1c were used for diagnosis, patients were classified to PD or T2DM if either the OGTT or HbA1c criteria were fulfilled.

## Data Availability

Not applicable.

## Abbreviations

ADA: American Diabetes Association
AIS: Acute ischemic stroke
ASPIS: Austrian Polyintervention Study to Prevent Cognitive Decline after Ischemic Stroke
FPG: Fasting plasma glucose
HbA1c: Glycated haemoglobin
IFG: Impaired fasting glucose
IGT: Impaired glucose tolerance
NIHSS: National Institutes of Health Stroke Scale
mRS: Modified Rankin Scale
OGTT: Oral glucose tolerance test
T2DM: Type 2 diabetes mellitus
2hPG: 2-h plasma glucose
WHO: World Health Organization
