# Supplementary material for: Comparison of oral glucose tolerance test and HbA1c in detection of disorders of glucose metabolism in patients with acute stroke
Source: Cardiovasc Diabetol. 2020 Dec 5;19:204. doi: 10.1186/s12933-020-01182-6 (PMC7719250; doi:10.1186/s12933-020-01182-6)
Supplement: Supplementary file 1 — Additional file 1: Table S1. Baseline characteristics of patients in different groups of evolution of glucose metabolism (GM) between baseline and 12 months follow up. Both, OGTT and HbA1c were used for diagnosis, patients were classified to PD or T2DM if either the OGTT or HbA1c criteria were fulfilled. [file 12933_2020_1182_MOESM1_ESM.docx]

|  | **Progression to T2DM or PD (n=19)** | **Stable GM (n=67)** | **Improved GM (n=16)** | **p** |
| --- | --- | --- | --- | --- |
| Sex, female, n (%) | 7 (36.8%) | 19 (28.4%) | 2 (12.5%) | p= 0.26 |
| Age, mean (SD) | 60.4 (12.1) | 61.8 (9.6) | 60.8 (7.9) | p = 0.778 |
| BMI, mean (SD) | 27.2 (4.1) | 27.7 (4.1) | 29.1 (3.5) | p = 0.441 |
| mRS before stroke=0, n (%) | 13 (81.3%) | 62 (95.4%) | 16 (100%) | p = 0.006 |
| NIHSS at admission, mean (SD) | 0.8 (0.86) | 1.7 (1.82) | 2.2 (2.1) | p = 0.063 |
| MMSE Baseline, mean (SD) | 28.3 (2) | 28.9 (1.2) | 28.6 (1.2) | P = 0.445 |
| Arterial Hypertension, n (%) | 15 (78.9%) | 53 (79.1%) | 10 (62.5%) | p = 0.42 |
| Hyperlipidemia, n (%) | 13 (68.4%) | 54 (80.6%) | 13 (81.3%) | p= 0.49 |
| Current smoker, n (%) | 5 (26.3%) | 16 (23.9%) | 5 (31.3%) | p = 0.84 |
| Atrial fibrillation, n (%) | 3 (15.8%) | 6 (9.0%) | 1 (6.3%) | p = 0.60 |
| Coronary heart disease, n (%) | 4 (21%) | 7(10.4%) | 0 (0%) | p = 0.11 |
| Previous stroke or TIA, n (%) | 3 (15.7%) | 8 (14%) | 3 (18.8%) | p = 0.69 |
| Multifactorial intervention, n (%) | 7 (36.8%) | 32 (47.8%) | 12 (75%) | p = 0.063 |

**Additional Table**  Baseline characteristics of patients in different groups of evolution of glucose metabolism (GM) between baseline and 12 months follow up. Both, OGTT and HbA1c were used for diagnosis, patients were classified to PD or T2DM if either the OGTT or HbA1c criteria were fulfilled.
